# The Place of Therapeutic Apheresis in Severe Immune‐Related Adverse Events: A Scoping Review of Literature

**DOI:** 10.1002/jca.70199

**Published:** 2026-10-28

**Authors:** C. Vuillet, O. Moranne, D. Daubin, X. Quantin, P. Palassin, T. Kanouni, A. T. J. Maria, the MonRIO network (Montpellier Réseau Immuno‐Oncologie), Q. Thomas, P. Witkowski Durand Viel, C. Coustal, S. Faure, L. Meunier, L. Hountondji, R. Altwegg, C. Lesage, A. Lamoureux, G. Mouterde, S. Fabre, G. Taieb, F. Esselin, X. Ayriginac, C. Travert, B. Roch, F. Roubille, I. Raingeard, M. Le Quintrec, K. Dessaix, F. Garo, O. Moranne, J‐L. Faillie, P. Palassin, K. Klouche, B. Jauzelon, S. Bisiou, P. Rozier, A. TJ Maria

**Affiliations:** ^1^ Internal Medicine and Clinical Immunology Department Montpellier University Hospital Montpellier France; ^2^ Nephrology, Dialysis, Apheresis Department IDESP Montpellier University, Nîmes University Hospital Nîmes France; ^3^ Intensive Care Unit Montpellier University Hospital Montpellier France; ^4^ Regional Institute of Cancer Montpellier France; ^5^ Medical Pharmacology and Toxicology Department Montpellier University Hospital Montpellier France; ^6^ Hematology and Apheresis Department Montpellier University Hospital Montpellier France; ^7^ IHU Immun4Cure, IRMB, Inserm U1183, Montpellier University Montpellier France

**Keywords:** cancer, immune checkpoint inhibitors, immune‐related adverse events, immunotherapy, immunotoxicity, plasma exchange, plasmapheresis

## Abstract

Immune checkpoint inhibitors (ICIs) have revolutionized the management of patients with cancer. However, they can induce breakdowns in immunologic tolerance, leading to autoimmune diseases, or immune related adverse events (irAEs). These irAEs can sometimes be life threatening, requiring aggressive management, such as immunosuppressant (IS) or even therapeutic apheresis (TA). TA, an extracorporeal circulation technique, may represent a therapeutic option for severe irAEs, reducing their severity and duration by removing ICIs, pathogenic antibodies or inflammatory cytokines, although its role in this situation remains underreported. We made scoping review to better identify the use of TA for managing severe irAEs. From January 2022 to August 2025, three databases were reviewed, for articles mentioning the use of TA in the event of immunotoxicity. Only case reports or case series were retained. A descriptive summary was produced in table forms. Between 2022 and 2025, 162 articles were identified, 34 articles concerning 42 patients treated for severe irAE via TA were included. The underlying cancers were mainly non‐small cell lung cancer and melanoma, and 78% of patients were treated with monotherapy anti‐PD(L)1. The main indications for TA were neurological (40.6%), overlap manifestations (23.8%), and hepatitis (21.4%), mainly highly severe. TA was most often used in rescue situations, that is, after failure of several lines of IS. Complications of TA were rare. Clinical improvement was reported in most patients, usually within days of TA, with 24% complete resolution, and 45% partial resolution. Despite revolutionizing cancer treatment, ICIs induce severe irAEs, with poorly defined therapeutic options. TA may be a therapeutic option for life threatening irAEs, with data in the literature assuming its effectiveness. However, details on its use remain poorly defined. Further studies are required to emphasize its use as in irAEs.

**Abbreviations**
ASFA: American Society for Apheresis
CIDP: chronic inflammatory demyelinating polyradiculoneuropathy
CS: corticosteroids
CTCAE: Common Terminology Criteria for Adverse Events
CTLA‐4: Cytotoxic T‐Lymphocyte Antigen 4
DFPP: double filtration plasmapheresis
DPMAS: double plasma molecular adsorption system
ENT: ear, nose, throat
ICIs: immune checkpoint inhibitors
irAE: immune related adverse event
IS: immunosuppressant
IVIg: intravenous immunoglobulin
LAG‐3: Lymphocyte Activation Gene‐3
MMF: mycophenolate mofetil
PD‐(L)1: Programmed Death‐(Ligand) 1
TA: therapeutic apheresis
TTP: thrombotic thrombocytopenic purpura

## 1 Introduction

Cancer immunotherapies, known as immune checkpoint inhibitors (ICIs), have revolutionized the management of cancer patients, with increasingly broad indications, resulting in sustainable oncological responses and long‐term survival even in patients with advanced‐stage diseases [1]. These ICIs were initially approved for metastatic cancers, especially melanoma, non‐small cell lung cancer, and kidney cancers, and rapidly gained approval for a wide range of cancer types, including earlier treatment settings such as adjuvant and neoadjuvant therapy [2].

ICIs are monoclonal antibodies, targeting PD‐1, the PD‐1 ligand (PD‐L1), CTLA‐4, and LAG3, designed to block key regulatory signals that dampen the immune response, restoring T cell–mediated anti‐tumor immunity [3]. Disrupting these key regulatory signals can lead to a breakdown in immune tolerance and trigger autoimmune manifestations affecting any organ, known as immune related adverse events (irAEs) [4].

Most patients will present at least one irAE [5], which can sometimes be severe or life‐threatening according to the organ involved: mainly cardiac and neurological damage. Although fatal irAEs are rare (0.5%–1.2% of all irAEs) [6], myocarditis or overlap syndromes combining myositis and/or myasthenia account for up to half of ICI‐related deaths [7, 8]. These rare but severe conditions require prompt, aggressive management, typically with pulses of high‐dose corticosteroids (CS) [9]. In cases of CS‐refractory disease, additional immunosuppressive strategies may be required, including intravenous immunoglobulins (IVIgs), Mycophenolate Mofetil (MMF), biologic agents such as anti‐TNF α, Rituximab, or therapeutic apheresis (TA) [5].

TA refers to several extracorporeal blood purification techniques, including therapeutic plasma exchange (TPE), immunoadsorption (IA), double filtration plasmapheresis (DFPP), and double plasma molecular adsorption system (DPMAS). TPE is a non‐selective technique that removes circulating plasma constituents through plasma replacement, whereas IA, DFPP, and DPMAS allow more selective removal of immunoglobulins, antibodies, or inflammatory mediators [10, 11]. These approaches may facilitate the elimination of circulating ICIs and immune mediators involved in severe irAEs.

The main indications for TA are reported and discussed in the ASFA guidelines updated every 3 years. In the 2023 updated recommendations, irAEs represent an emerging indication for TA, though the evidence remains low (Category III, Grade 2C) [12].

The rationale for TA in this indication lies in accelerating the clearance of ICI antibodies, characterized by their long half‐life IgG, high molecular weight, and low extravascular volume of distribution, which can be directly removed by TA [13]. Additionally, TA may help attenuate severe irAEs by removing inflammatory cytokines or pathogenic autoantibodies, potentially reducing both the severity and duration of symptoms [14].

Nevertheless, the precise role of TA in irAE management remains unclear. The available data are scarce, often limited to cases with severe organ involvement (neurological, muscular, cardiac, or renal). Reports in the literature provide little detail regarding TA procedures, patient outcomes, or comparisons with patients managed without TA.

This review follows the one published by Onwuemene et al. in 2022 [15], which identified 81 studies involving 111 patients between 2014 and 2022 treated with apheresis for irAEs. In that study, neurological disorders were the most common (53% of conditions treated), with a significant proportion of overlap syndromes (24%; *N* = 16). There were variable, but often positive, clinical outcomes, and a good overall tolerance profile for plasmapheresis.

The present review builds upon the work by Onwuemene et al. and incorporates newly published reports and provides a contemporary overview of the use of TA for severe immune‐related adverse events.

## 2 Materials and Methods

### 2.1 Study Objective

This study was conducted as a scoping review aiming to evaluate the existing evidence of TA for managing irAEs.

This approach was chosen due to the limited, heterogeneous, and predominantly descriptive nature of the available literature. The aim was to describe patient characteristics, irAE features, technical modalities of TA, clinical outcomes, and oncological evolution reported in the literature.

In the present review, TA was defined as any extracorporeal blood purification technique used to remove circulating ICIs and/or pathogenic immune mediators, including therapeutic plasma exchange (TPE), DFPP, and DPMAS. Other apheresis modalities, such as extracorporeal photopheresis (ECP), were not within the scope of this review. No case using IA was ultimately identified in the included literature.

Given the study design, which is a review of literature, there was no need for ethical approval or informed consent.

### 2.2 Study Design and Data Collection

We search three different databases: PubMed, Google Scholar, and Cochrane, aiming to identify relevant studies published between January 2022 and August 2025.

The search strategy combined keywords and their synonyms: (“plasmapheresis” OR “plasma exchange” OR “therapeutic apheresis”) AND (“immune‐related adverse events” OR “irAEs” OR “immunotoxicity”) AND (“immune checkpoint inhibitors” OR “ICI”).

Search terms were adapted for each database to ensure sensitivity of retrieval. Only articles in English or French and available in full text were included.

### 2.3 Inclusion and Exclusion Criteria

Eligible sources included case reports, case series, and observational studies reporting the use of plasmapheresis for immune‐mediated adverse effects related to ICIs.

All studies involving TA for indications unrelated to the use of ICIs, narrative reviews without original data, studies lacking sufficient clinical information for data extraction, preprints, and publications not written in French or English were excluded from the study.

### 2.4 Selection Process

All records identified through the search strategy were screened based on titles and abstracts. Full‐text articles were assessed for eligibility when relevance was suspected.

The selection process was documented using a PRISMA flow diagram. Study selection was performed by one reviewer, and any discrepancies were resolved through discussion with a second reviewer to reach consensus.

### 2.5 Data Extraction

Data were extracted using a predefined standardized form. The following variables were collected: patient demographics (age, sex), cancer type, ICI regimen, characteristics of irAEs (type, severity graded using Common Terminology Criteria for Adverse Events [CTCAE], time to onset), and details of TA (timing of initiation, number of sessions, frequency, modality, replacement fluids, anticoagulation strategy, vascular access, and technical devices used).

Additional variables included concomitant immunosuppressive treatments, adverse events related to TA, clinical response, time to resolution of irAEs, and oncological outcomes at the last follow‐up.

Outcomes were categorized as complete resolution, partial improvement, no response, or death, according to the description provided in the original reports. When outcome reporting was insufficiently detailed, the classification was based on the authors' assessment of clinical and/or biological evolution.

### 2.6 Data Analysis

A descriptive summary of the studies included was made. Continuous variables were summarized using medians or ranges when available, whereas categorical variables were reported as frequencies and proportions. Given the heterogeneity and descriptive nature of the literature included, no quantitative meta‐analysis was conducted.

### 2.7 Quality Assessment/Risk of Bias

As this study was designed as a scoping review, no formal risk‐of‐bias assessment was systematically applied. Nevertheless, the methodological quality and reporting completeness of the included studies were critically considered during data extraction. To guide this appraisal, the Joanna Briggs Institute (JBI) Critical Appraisal Checklists for Case Reports and Case Series were used as a methodological framework, according to the study design [16], and uncertainties were discussed with a second reviewer. Particular attention was paid to the reporting of patient characteristics, diagnostic procedures, therapeutic interventions, clinical outcomes, follow‐up data, and TA procedural details.

## 3 Results

As summarized in the PRISMA diagram (Figure 1), three databases were reviewed: PubMed, Cochrane Library, and Google Scholar, selecting case reports or case series reporting the confirmed use of TA in the context of immunotoxicity. Between January 2022 and August 2025, 162 articles were identified, and finally 34 publications were included, representing 42 patients.

**FIGURE 1:**
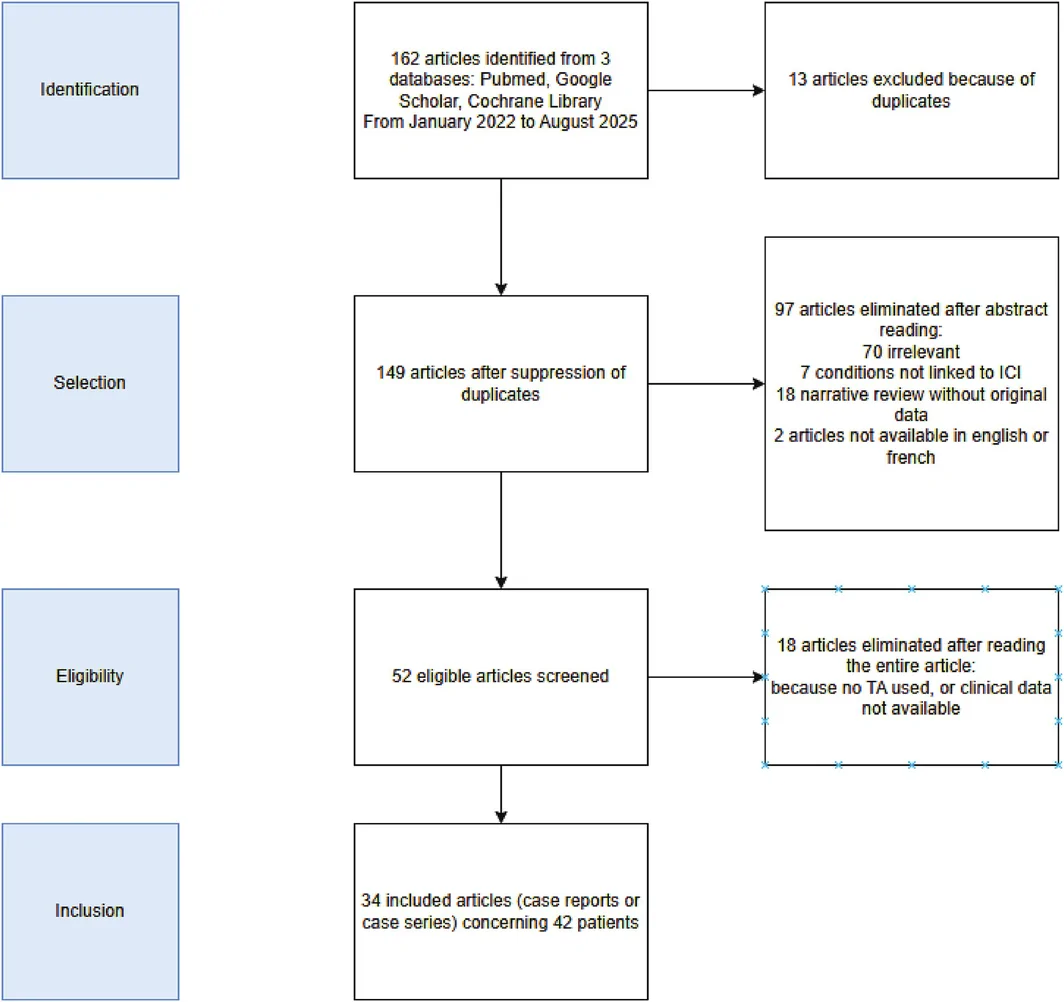
PRISMA diagram.

### 3.1 Population Characteristics

Table 1 summarizes the patients' demographic and oncological characteristics.

**TABLE 1:** Basic characteristics of patients with irAEs treated by TA.

Characteristics	*N* = 42
Male Gender	30/42 (71.4%)
Median age at onset of irAE (min–max)	70.5 (30–87)

Cancer type	
Lung	8 (19%)
Melanoma	8 (19%)
Kidney	5 (11.9%)
Bladder	4 (9.5%)
ENT	3 (7.1%)
Esophagus	2 (4.8%)
Gastric	2 (4.8%)
Thymus	2 (4.8%)
Hodgkin's lymphoma	2 (4.8%)
Breast	1 (2.4%)
Colon	1 (2.4%)
Squamous cell carcinoma	1 (2.4%)
Vulvar squamous cell carcinoma	1 (2.4%)
Hepatocellular carcinoma	1 (2.4%)
Sarcoma	1 (2.4%)

Type of ICI	
Monotherapy anti PD(L)‐1	33 (78.6%)
Combi‐therapy anti PD1 + anti CTLA4	9 (21.4%)

ICI molecule	*N* = 42
Anti‐PD1	27 (64.3%)
Pembrolizumab	11
Nivolumab	7
Camrelizumab	2
Sintilimab	4
Tirelizumab	1
Cemiplimab	1
Toripalimab	1
Anti‐PD L 1	6 (14.3%)
Durvalumab	4
Atezolizumab	2
Combined immunotherapy	9 (21.4%)
Nivolumab + Ipilimumab	8
Nivolumab + anti‐LAG3	1

Median number of injections (min‐max)	2 (1–8)
Median number of days (min‐max) between last ICI injection and onset of irAE	14 (1–300)

Associated treatment	
Chemotherapy	5 (11.4%)
Radiotherapy	2 (4.5%)
Bevacizumab (anti‐VEGF)	1 (2.3%)
Tiragolumab (anti‐TIGIT)	1 (2.3%)

The underlying cancers were mainly non‐small cell lung cancer and melanoma, followed by renal, bladder, and ENT (ear, nose, throat) cancers. Concerning the ICI they received, 78% of patients received monotherapy with anti‐PD(L)1, and 22% received combined‐therapy with anti‐PD(L)1 and anti‐CTLA4 or LAG3.

Characteristics of irAEs are described in Table 2. The main indications for TA were neurological (40.6%) and overlap manifestations including neuromuscular and heart damages (23.8%), but there was also a significant proportion of hepatitis (21.4%) and other organ damages (renal, pulmonary, hematologic involvement), mainly with a high grade of severity, that is, grade ≥ 3 CTCAE. TA was almost indicated in rescue situation, that is, after failure of previous lines of immunosuppressants (IS). When specified, patients were mostly managed in intensive care units.

**TABLE 2:** Characteristics of irAEs and management.

irAEs characteristics	Patients treated with TA *N* = 42
	*N* (%) or median (min–max)
Type of irAE: Number of events	
Neurological	17
Myasthenia [17, 18, 19, 20, 21, 22]	6
Myositis [23, 24, 25, 26, 27]	5
Encephalitis [28, 29, 30]	3
Myelitis [31]	1
CIDP [32]	1
Optic neuropathy [33]	1
Overlap	10
Myositis, myasthenia, myocarditis [34, 35, 36, 37, 38]	6
Myositis + myasthenia [22, 39, 40]	3
Myositis + myocarditis [41]	1
Myocarditis [42]	1
Hepatitis [43, 44, 45]	9
TTP [46]	1
Relapse of lupus nephritis [47]	1
Rapid glomerulonephritis [48]	1
Interstitial pneumonitis [49]	1
Acute lung transplant rejection [50]	1
CTCAE grade	
3	5 (11.9%)
4	21 (50%)
5	1 (2.4%)
Unspecified	15 (35.7%)
Other characteristics	
Associated other irAE (Grades 1–2)	3
Associated auto‐antibody	8
Care department	
Intensive care unit	14 (33.3%)
Neurology	1 (2.4%)
Nephrology	1 (2.4%)
Pneumology	1 (2.4%)
Unspecified	25 (59.5%)
Treatment prior to TA	
Median number of IS (min–max)	1 (1–4)
Corticosteroids	39 (92.8%)
Other immunosuppressants	24 (57.1%)
IVIg	14
Mycophenolate mofetil	3
Infliximab	2
Tocilizumab	2
Rituximab	1
Abatacept	1
Methotrexate	1

Abbreviation: CIDP, chronic inflammatory demyelinating polyradiculoneuropathy.

When specified, pathogenic autoantibodies were found for eight patients: GABAR1 and GAD 65, anti‐skeletal muscle and anti‐cardiac muscle, anti‐DNA and anti‐Sm, anti‐titin, anti‐GBM, anti‐AChR, anti‐ADAMTS13, anti‐Kv1.4. Five patients presenting with myasthenia received pyridostigmine.

Few details were available regarding the technical and practical implementation of TA. Detailed procedural characteristics were reported in only 10/42 patients (23.8%), whereas information regarding vascular access, anticoagulation, replacement fluid, or device type was available for less than 5% of cases. Four patients were treated by both single plasma exchange and DPMAS, four patients were treated with single plasma exchange, and two patients were treated by DFPP. Patients received an average of 4 sessions (min–max 1–14). Fourteen patients received other IS after TA, including 50% IVIg, and 50% Rituximab, three received MMF and one tacrolimus.

Concerning tolerance, complications with TA were rare in these reports (one pulmonary edema and one pneumonia), with no serious events directly linked to the procedure being described.

Patient outcomes, with irAE resolution are described in Table 3. Clinical improvement (neurological or cardiac recovery, normalization of muscle, cardiac, renal, or hepatic biomarkers) was reported for most patients, usually within days of TA, with 24% complete resolution and 45% partial resolution, with some patients presenting neurological motor sequelae or keeping cardiac or renal enzyme levels above normal range, for example.

**TABLE 3:** Patient outcomes.

Part 1				
irAE category	*N* = 42	Complete resolution, *n* (%)	Partial resolution, *n* (%)	No resolution, *n* (%)
Myasthenia	6	0	4 (66.7%)	2 (33.3%)
Myositis	5	1 (20%)	3 (60%)	1 (20%)
Encephalitis	3	2 (66.7%)	1 (33.3%)	
Myelitis	1		1 (100%)	
CIDP	1		1 (100%)	
Optic neuropathy	1	1 (100%)		
Overlap	10	4 (40%)	1 (10%)	5 (50%)
Myocarditis	1			1 (100%)
Hepatitis	9	1 (11.1%)	8 (88.9%)	
TTP	1			1 (100%)
Lupus nephritis	1			1 (100%)
Glomerulonephritis	1			1 (100%)
Interstitial pneumonitis	1			1 (100%)
Acute lung transplant rejection	1	1 (100%)		

Part 2				
ICI regimen	*N* = 42	Complete resolution, *n* (%)	Partial resolution, *n* (%)	No resolution, *n* (%)
Mono‐therapy	33	8 (24.2%)	17 (51.6%)	8 (24.2%)
Combined therapy	9	2 (22.2%)	2 (22.2%)	5 (55.6%)

All patients reported as having hepatic irAEs experienced an improvement following TA, whereas outcomes were more heterogeneous among neurological and cardiac toxicities. The median time to partial or complete resolution of irAE was 11 days but was not evenly reported (only 8 articles mentioned this time).

We noted 12 deaths (28% of patients). Six were due to the irAE itself (three respiratory failures due to myasthenia, cardiac failure due to myocarditis, evolution of interstitial pneumonitis, cerebral infarction linked to TTP), three due to infectious pneumonitis, two due to cancer progression, one of unknown cause. Oncological outcomes should be cautiously interpreted, as follows: follow‐up duration was inconsistently reported across studies and was unavailable for 45% of cases. About 15% of patients had a complete or partial oncological response at their last visit (*n* = 6), four patients had a stable disease, and three had a progressive disease.

## 4 Discussion

To summarize, our updated review of the literature confirms the usage profile of TA observed in the previous review of 2022; however, there are a few differences.

In this review, the underlying malignancies were predominantly non‐small cell lung cancer and melanoma, consistent with the broader epidemiology of ICI use. As expected, most patients received anti‐PD(L)1 monotherapy, whereas a smaller proportion received combination regimens with anti‐CTLA4 or LAG‐3.

Overall, our findings are consistent with the spectrum of severe irAEs treated by TA reported by Onwuemene et al. [15]. Neuromuscular and cardiac toxicities remained the most commonly reported indications. However, the more recent literature included a greater proportion of hepatic and renal toxicities treated by TA. Indeed, compared with the review by Onwuemene et al., a higher proportion of hepatic toxicities was observed in the more recently published literature (21% vs. 4%). Although the reason remains uncertain, this observation may reflect the growing use of ICIs, earlier, improved recognition of less common irAEs, and a reporting bias inherent to the available case‐based literature.

In nearly all cases, TA was implemented as rescue therapy, after failure of standard immunosuppressive regimens such as CS, IVIg, or MMF. Patients were often managed in intensive care units, underlining the severity of clinical presentations. Interestingly, autoantibodies were identified in a subset of cases, supporting the hypothesis that autoreactive humoral responses may contribute to the pathophysiology of certain irAEs and provide a rationale for TA.

The key technical parameters of TA—including modality, timing of initiation, number and frequency of sessions, replacement fluids, anticoagulation strategies, and vascular access—were inconsistently reported across studies. This lack of standardized and detailed reporting limits the reproducibility of the intervention and reduces the clinical applicability of the findings.

Nevertheless, TA was generally well tolerated, with no major complications directly attributable to the procedure being described. Clinical outcomes were encouraging, with almost 70% of patients experiencing at least partial resolution of irAEs, often within days of starting TA. Still, a significant number of patients died, reflecting the gravity of underlying diseases and the severity of irAEs rather than TA itself.

The results of this study are consistent with previous reviews and confirm that TA mainly remains a rescue option in the context of severe, refractory irAEs. However, its potential benefit in other manifestations, particularly hepatitis and renal involvement, deserves further attention. The scarcity of data on procedural parameters and oncologic follow‐up remains a critical gap in the literature.

A potential benefit from TA could also be corticosteroid (CS) sparing. Indeed, the use of high dose or long duration CS is known to cause several adverse events, including metabolic complications (diabetes mellitus, hypertension). Furthermore, in a recent study, Verheijden et al. [51] have shown that high doses of CS may lead to detrimental effects on overall survival. Indeed, although CS and other IS are required to prevent mortality in irAEs, the initial high peak dose of CS and the use of secondary IS are independently associated with poor survival in patients with advanced melanoma treated with ICIs. Considering the results of this study, TA may represent an option for avoiding high doses of CS or several second‐line IS.

Furthermore, the possibility of introducing TA earlier in the management of selected irAEs is emerging as an area of clinical interest, although further prospective studies are required to clarify its role, establish standardized protocols, and assess long‐term outcomes.

This review has several limitations. First, the available evidence is limited to case reports and small case series, which are inherently subject to publication and reporting bias. Second, the overall number of patients remains small, so robust conclusions regarding efficacy and safety must be avoided. Third, substantial heterogeneity was observed in cancer types, ICIs, irAE presentations, concomitant immunosuppressive therapies, and TA modalities. Fourth, certain important procedural characteristics of TA—including replacement fluids, anticoagulation regimens, vascular access, exchanged plasma volume, and device specifications—were missing, limiting reproducibility and comparison across studies. Fifth, outcome definitions and follow‐up durations were inconsistently reported, preventing standardized assessment of both irAE resolution and oncological outcomes. Finally, the concomitant use of CS, IVIg, and other immunosuppressive therapies limits the ability to determine the specific contribution of TA to clinical improvement.

The findings of this review should therefore be interpreted with caution. Indeed, it should be noted that irAEs are a rare, recently identified event, with treatment recommendations that are still inconsistent and relatively new, which also explains the low level of evidence in the literature.

Further well‐designed studies with larger sample sizes and standardized methodologies, including more comprehensive reporting of therapeutic procedures, are required to strengthen the evidence on this subject.

## 5 Conclusion

Although ICIs have revolutionized cancer treatment, they may lead to irAEs, sometimes severe or fatal, with therapeutic options which are still poorly defined. Based on the currently available literature, TA appears to be a promising adjunctive treatment option in selected patients with severe irAEs, particularly when it is necessary to quickly remove circulating ICIs or immune mediators.

However, the available evidence remains limited to case reports and small case series, with substantial heterogeneity in patient characteristics, irAE presentations, concomitant immunosuppressive therapies, and apheresis procedures. Furthermore, procedural details are frequently underreported, limiting the reproducibility and interpretation of published outcomes.

Thus, although the reported clinical responses are encouraging, the precise role, optimal timing, and technical modalities of TA in irAEs management remain to be defined. Prospective studies and standardized reporting of apheresis procedures are required to better characterize its efficacy, safety, and place within current treatment strategies.

## Funding

The authors have nothing to report.

## Conflicts of Interest

The authors declare no conflicts of interest.

## Data Availability

The data that support the findings of this study are available from the corresponding author upon reasonable request.

## References

[1] L. B. Kennedy and A. K. S. Salama , “A Review of Cancer Immunotherapy Toxicity,” CA: a Cancer Journal for Clinicians 70, no. 2 (2020): 86–104, 10.3322/caac.21596. 31944278

[2] R. K. Vaddepally , P. Kharel , R. Pandey , R. Garje , and A. B. Chandra , “Review of Indications of FDA‐Approved Immune Checkpoint Inhibitors Per NCCN Guidelines With the Level of Evidence,” Cancers 12, no. 3 (2020): 3, 10.3390/cancers12030738. PMC7140028 32245016

[3] A. D. Waldman , J. M. Fritz , and M. J. Lenardo , “A Guide to Cancer Immunotherapy: From T Cell Basic Science to Clinical Practice,” Nature Reviews. Immunology 20, no. 11 (2020): 651–668, 10.1038/s41577-020-0306-5. PMC7238960 32433532

[4] D. M. Pardoll , “The Blockade of Immune Checkpoints in Cancer Immunotherapy,” Nature Reviews. Cancer 12, no. 4 (2012): 252–264, 10.1038/nrc3239. PMC4856023 22437870

[5] J. R. Brahmer , C. Lacchetti , B. J. Schneider , et al., “Management of Immune‐Related Adverse Events in Patients Treated With Immune Checkpoint Inhibitor Therapy: American Society of Clinical Oncology Clinical Practice Guideline,” Journal of Clinical Oncology 36, no. 17 (2018): 1714–1768, 10.1200/JCO.2017.77.6385. PMC6481621 29442540

[6] D. Y. Wang , J. E. Salem , J. V. Cohen , et al., “Fatal Toxic Effects Associated With Immune Checkpoint Inhibitors: A Systematic Review and Meta‐Analysis,” JAMA Oncology 4, no. 12 (2018): 1721–1728, 10.1001/jamaoncol.2018.3923. PMC6440712 30242316

[7] A. Cozma , N. D. Sporis , A. L. Lazar , et al., “Cardiac Toxicity Associated With Immune Checkpoint Inhibitors: A Systematic Review,” International Journal of Molecular Sciences 23, no. 18 (2022): 18, 10.3390/ijms231810948. PMC9502843 36142866

[8] R. Pathak , A. Katel , E. Massarelli , V. M. Villaflor , V. Sun , and R. Salgia , “Immune Checkpoint Inhibitor–Induced Myocarditis With Myositis/Myasthenia Gravis Overlap Syndrome: A Systematic Review of Cases,” Oncologist 26, no. 12 (2021): 1052–1061, 10.1002/onco.13931. PMC8649039 34378270

[9] S. Champiat , O. Lambotte , E. Barreau , et al., “Management of Immune Checkpoint Blockade Dysimmune Toxicities: A Collaborative Position Paper,” Annals of Oncology 27, no. 4 (2016): 559–574, 10.1093/annonc/mdv623. 26715621

[10] A. P. Sanchez , R. Cunard , and D. M. Ward , “The Selective Therapeutic Apheresis Procedures,” Journal of Clinical Apheresis 28, no. 1 (2013): 20–29, 10.1002/jca.21265. 23420592

[11] O. Moranne , C. Roux , I. M. Ion , and S. Chkair , “Therapeutic Plasmapheresis Procedures: An Alternative to the Disruption of the Supply of Polyvalent Immunoglobulin in Autoimmune Pathologies. Medico‐Economic Study,” Néphrologie & Thérapeutique 18, no. 3 (2022): 172–179, 10.1016/j.nephro.2022.02.004. 35644771

[12] L. Connelly‐Smith , C. R. Alquist , N. A. Aqui , et al., “Guidelines on the Use of Therapeutic Apheresis in Clinical Practice – Evidence‐Based Approach From the Writing Committee of the American Society for Apheresis: The Ninth Special Issue,” Journal of Clinical Apheresis 38, no. 2 (2023): 77–278, 10.1002/jca.22043. 37017433

[13] M. Centanni , D. J. A. R. Moes , I. F. Trocóniz , J. Ciccolini , and J. G. C. van Hasselt , “Clinical Pharmacokinetics and Pharmacodynamics of Immune Checkpoint Inhibitors,” Clinical Pharmacokinetics 58, no. 7 (2019): 835–857, 10.1007/s40262-019-00748-2. PMC6584248 30815848

[14] T. R. Katsumoto , K. L. Wilson , V. K. Giri , et al., “Plasma Exchange for Severe Immune‐Related Adverse Events From Checkpoint Inhibitors: An Early Window of Opportunity?,” Immunotherapy Advances 2, no. 1 (2022): ltac012, 10.1093/immadv/ltac012. PMC9257781 35814850

[15] O. A. Onwuemene , C. I. Nnoruka , C. J. Patriquin , and L. S. Connelly‐Smith , “Therapeutic Plasma Exchange in the Management of Immune Checkpoint inhibitor‐associated Immune‐Related Adverse Effects: A Review,” Transfusion 62, no. 11 (2022): 2370–2390, 10.1111/trf.17114. 36134464

[16] “4. Systematic Reviews of Effectiveness [Internet],” (2025), https://jbi‐global‐wiki.refined.site/space/MANUAL/355827955/jbi‐global‐wiki.refined.site.

[17] L. Montag , R. Piver , A. Vidalin , M. Johnson , B. Rungruang , and R. Higgins , “Pembrolizumab‐Induced Myasthenia Gravis: Two Patients' Experiences,” Gynecologic Oncology Reports 54 (2024): 101453, 10.1016/j.gore.2024.101453. PMC11301092 39108615

[18] T. I. Kosick , K. Patel , J. Jasinski , and B. Dada , “A Case of Pembrolizumab‐Induced Myasthenia Gravis,” Cureus 9, no. 15 (2023): e45455, 10.7759/cureus.45455. PMC10583611 37859893

[19] A. Schwab , M. Assaad , R. Hamadi , et al., “Pembrolizumab‐Induced Myasthenia Gravis and Myositis: Literature Review on Neurological Toxicities of Programmed Death Protein 1 Inhibitors,” Journal of Medical Cases 13, no. 11 (2022): 530–535, 10.14740/jmc4008. PMC9728146 36506763

[20] J. S. Thyagarajan , M. H. Almomani , N. Thatikonda , C. Patel , and X. Fang , “A Case of Myasthenia Gravis: A Paraneoplastic Syndrome or an Immune‐Related Disorder?,” Cureus 17, no. 2 (2025): e78640, 10.7759/cureus.78640. PMC11890160 40062097

[21] Y. Kitazaki , O. Yamamura , K. Usui , et al., “Anti‐Kv1.4 Antibody‐Positive Nivolumab‐Induced Myasthenia Gravis and Myositis Presenting With Bilateral Ptosis and Demonstrating Different Pathophysiologies,” Internal Medicine 62, no. 20 (2023): 3013–3020, 10.2169/internalmedicine.0739-22. PMC10641203 37839874

[22] Z. Elavia , V. Jimenez , R. Lockhart , A. Muha , and M. Kazamel , “Myositis/Myasthenia Gravis Caused by Immune Checkpoint Inhibition: A Report of Two Cases and a Brief Review of the Literature,” Medicine International (London) 5, no. 1 (2025): 11, 10.3892/mi.2024.210. PMC11668140 39720481

[23] N. Katyal , T. R. Katsumoto , K. J. Ramachandran , M. Yunce , and S. Muppidi , “Plasma Exchange in Patients With Myositis due to Immune Checkpoint Inhibitor Therapy,” Journal of Clinical Neuromuscular Disease 25, no. 2 (2023): 89–93, 10.1097/CND.0000000000000457. PMC10645099 37962196

[24] G. Hong , H. Zhao , Y. Yin , et al., “Sintilimab‐Induced Inflammatory Myopathy in a Patient With Esophageal Cancer: A Case Report,” Frontiers in Immunology 14 (2023): 1253463, 10.3389/fimmu.2023.1253463. PMC10619899 37920461

[25] T. Baigi , E. N. Brown , R. M. De La Torre , and F. I. Abu‐Shahin , “Atezolizumab‐Associated Myositis in a Patient With Unresectable Hepatocellular Carcinoma,” Journal of Oncology Pharmacy Practice 29, no. 7 (2023): 1757–1761, 10.1177/10781552231180876. 37282559

[26] J. R. Walder , S. A. Faiz , S. Tummala , et al., “Diaphragmatic Myopathy Associated With Dual Immune Checkpoint Inhibitors in a Patient With Renal Cell Carcinoma,” Journal for Immunotherapy of Precision Oncology 7, no. 4 (2024): 304–307, 10.36401/JIPO-23-45. PMC11541929 39553443

[27] N. A. Abdeljabbar , S. Genovese , M. B. Haradwala , and M. Sivaraman , “Immune Checkpoint Inhibitor‐Induced Myositis Causing Severe Ptosis: A Case Report,” Cureus 16, no. 11 (2024): e73444, 10.7759/cureus.73444. PMC11633727 39664141

[28] T. T. Moss , K. Stavem , A. Aandahl , et al., “Case Report: Limbic Encephalitis Following Treatment With Durvalumab for Small‐Cell Lung Cancer,” Frontiers in Immunology 14 (2023): 1278761, 10.3389/fimmu.2023.1278761. PMC10613972 37908347

[29] M. W. Buckley , A. Balaji Warner , J. Brahmer , et al., “Immune‐Related Encephalitis After Immune Checkpoint Inhibitor Therapy,” Oncologist (2024): oyae186, 10.1093/oncolo/oyae186. PMC11783331 39066587

[30] G. Civardi , A. Medioli , C. Braghieri , et al., “Autoimmune Encephalitis Following Checkpoint Inhibitor Therapy in a Patient With Metastatic Melanoma in Complete Remission,” Medicina (Kaunas, Lithuania) 60, no. 5 (2024): 728, 10.3390/medicina60050728. PMC11123015 38792910

[31] S. Chatterton , S. Xi , J. X. Jia , et al., “Case Series: Immune Checkpoint Inhibitor‐Induced Transverse Myelitis,” Frontiers in Neurology 14 (2023): 1130313, 10.3389/fneur.2023.1130313. PMC9989185 36895912

[32] O. A. Tikhonova , Т. О. Алексеевна , D. S. Druzhinin , et al., “Chronic Inflammatory Demyelinating Polyneuropathy Induced by Immune Checkpoint Inhibitors: Case Reports,” Annals of Clinical and Experimental Neurology 18, no. 1 (2024): 98–104, 10.54101/ACEN.2024.1.11.

[33] E. Daetwyler , A. Zippelius , P. Meyer , and H. Läubli , “Pembrolizumab‐Induced Optic Neuropathy – A Case Report,” Frontiers in Immunology 14 (2023): 1171981, 10.3389/fimmu.2023.1171981. PMC10203429 37228591

[34] H. Kamikawa , Y. Matsukawa , H. Nishii , et al., “A Case of Severe Immune‐Related Adverse Events, Myocarditis With Myositis, and Myasthenia Gravis Overlap Syndrome Following Adjuvant Nivolumab Administration for Muscle‐Invasive Bladder Cancer,” Nagoya Journal of Medical Science 87, no. 1 (2025): 156–162, 10.18999/nagjms.87.1.156. PMC12003993 40255993

[35] A. Sánchez‐Camacho , A. Torres‐Zurita , L. Gallego‐López , et al., “Management of Immune‐Related Myocarditis, Myositis and Myasthenia Gravis (MMM) Overlap Syndrome: A Single Institution Case Series and Literature Review,” Frontiers in Immunology 16 (2025): 1597259, 10.3389/fimmu.2025.1597259. PMC12095175 40406130

[36] B. Inan , U. Duzgun , Z. Ergul‐Ulger , et al., “Nivolumab‐ and Ipilimumab‐Induced Myositis, Myasthenia Gravis, and Myocarditis in a Patient With Metastatic Melanoma,” Anti‐Cancer Drugs 36, no. 7 (2025): 600–605, 10.1097/CAD.0000000000001727. 40343457

[37] S. Saito , S. Hontsu , J. Hiraoka , et al., “A Rare Case of Overlapping Durvalumab‐Induced Myositis, Takotsubo‐Like Morphological Changes Caused by Myocarditis, and Myasthenia Gravis,” Internal Medicine 63, no. 23 (2024): 3209–3215, 10.2169/internalmedicine.3028-23. PMC11671183 38658343

[38] Y. Saishu , T. Yoshida , Y. Seino , and T. Nomura , “Nivolumab‐Related Myasthenia Gravis With Myositis Requiring Prolonged Mechanical Ventilation: A Case Report,” Journal of Medical Case Reports 16, no. 1 (2022): 61, 10.1186/s13256-022-03286-x. PMC8842808 35152911

[39] C. Gosser , A. Al Bawaliz , W. Bahaj , J. Chesney , and S. Ranjan , “Immune Checkpoint Inhibitor‐Induced Myositis/Myasthenia Gravis Overlap,” Cureus 15, no. 11 (2023): e49007, 10.7759/cureus.49007. PMC10726830 38111441

[40] A. Hutchinson , D. Eskens , A. Chan , A. Bhargava , and R. Bycroft , “Managing Suspected Myasthenia Gravis and Myositis Induced by Pembrolizumab in a Jehovah's Witness Sarcoma Patient,” Journal of Oncology Pharmacy Practice 30, no. 5 (2024): 950–953, 10.1177/10781552241240734. 38523425

[41] S. B. Barker and A. Adewuyi , “Rapid‐Onset Myositis and Myocarditis Following Dual Immune Checkpoint Inhibitor Therapy With Nivolumab and Ipilimumab for Gastric Adenocarcinoma: A Case Report,” Cureus 17, no. 5 (2025): e84121, 10.7759/cureus.84121. PMC12165631 40519473

[42] C. Wang , B. Zhong , J. He , and X. Liao , “Immune Checkpoint Inhibitor Sintilimab‐Induced Lethal Myocarditis Overlapping With Myasthenia Gravis in Thymoma Patient: A Case Report,” Medicine (Baltimore) 102, no. 15 (2023): e33550, 10.1097/MD.0000000000033550. PMC10101245 37058040

[43] Y. Liu , L. Zheng , W. Xu , et al., “Efficacy of Plasma Exchange and a Double‐Plasma Molecular Absorption System for Treating Immune Checkpoint Inhibitor‐Related Hepatitis,” Hepatitis Monthly (2023): 23, https://brieflands.com/articles/hepatmon‐137153#abstract.

[44] T. Renault , L. Meunier , and C. Monet , “Acute Liver Failure Following Immune Checkpoint Inhibitors,” Clinics and Research in Hepatology and Gastroenterology 47, no. 8 (2023): 102203, 10.1016/j.clinre.2023.102203. 37660741

[45] T. Wei , Z. Wang , and X. Liu , “Adverse Reactions and Efficacy of Camrelizumab in Patients With Lung Adenocarcinoma With High PD‐L1 Expression: A Case Report,” Medicine (Baltimore) 102, no. 7 (2023): e32731, 10.1097/MD.0000000000032731. PMC9935992 36800626

[46] W. J. Mullally , F. J. Cooke , I. M. Crosbie , et al., “Case Report: Thrombotic‐Thrombocytopenic Purpura Following Ipilimumab and Nivolumab Combination Immunotherapy for Metastatic Melanoma,” Frontiers in Immunology 13 (2022): 871217, 10.3389/fimmu.2022.871217. PMC9067158 35514990

[47] Z. Zhou , Q. Zhang , and X. Wang , “Relapse of Lupus Nephritis Induced by Nivolumab in an 84‐Year‐Old Patient With Oral Cancer: A Case Report,” Journal of International Medical Research 51, no. 9 (2023): 3000605231202140, 10.1177/03000605231202140. PMC10541768 37773731

[48] K. Ishiga , R. Kobayashi , T. Kanaoka , et al., “Two Acute Kidney Injury Episodes After ICI Therapy: A Case Report,” CEN Case Reports 13, no. 5 (2024): 408–415, 10.1007/s13730-024-00855-5. PMC11444024 38453804

[49] Q. Li , M. Liu , Y. Liu , et al., “Lethal Immune‐Related Pneumonitis After Durvalumab Therapy for Small Cell Lung Cancer: A First Case in China,” Case Reports in Oncology 17, no. 1 (2024): 673–680, 10.1159/000538429. PMC11249548 39015639

[50] J. A. Wali , M. Yunce , and N. Narula , “Successful Use of Therapeutic Plasma Exchange for the Management of Acute Lung Transplant Rejection Secondary to Immune Checkpoint Inhibitor Therapy,” Transfusion and Apheresis Science 63, no. 6 (2024): 104024, 10.1016/j.transci.2024.104024. 39486166

[51] R. J. Verheijden , F. H. Burgers , J. C. Janssen , et al., “Corticosteroids and Other Immunosuppressants for Immune‐Related Adverse Events and Checkpoint Inhibitor Effectiveness in Melanoma,” European Journal of Cancer 207 (2024): 114172, 10.1016/j.ejca.2024.114172. 38905818

